# Disseminated Saprochaete capitata Fungemia in Acute Myeloid Leukemia: A Case Report and Literature Review From the United Arab Emirates

**DOI:** 10.7759/cureus.85898

**Published:** 2025-06-13

**Authors:** Aqeel Saleem, Zaid H Al Hassani, Ali Al Hassani

**Affiliations:** 1 Infectious Disease, Sheikh Tahnoon Medical City, Al Ain, ARE; 2 College of Medicine, University of Sharjah, Sharjah, ARE; 3 Internal Medicine, Tawam Hospital, Al Ain, ARE

**Keywords:** acute myeloid leukemia, fungemia, geotrichum capitatum, invasive fungal infection, neutropenia, saprochaete capitata, united arab emirates

## Abstract

*Saprochaete capitata* (formerly *Geotrichum capitatum*) is a rare fungal pathogen that predominantly affects immunocompromised individuals, particularly those with hematologic malignancies and prolonged neutropenia. We report a rare case of S. capitata fungemia in a 49-year-old male newly diagnosed with acute myeloid leukemia (AML), managed in the United Arab Emirates. Despite prophylactic fluconazole during induction chemotherapy, the patient developed persistent febrile neutropenia. Blood cultures drawn from a peripherally inserted central catheter (PICC) grew yeast, later identified as *S. capitata* via biochemical profiling and carbohydrate assimilation testing. The isolate was resistant to fluconazole and echinocandins but susceptible to amphotericin B and voriconazole. Combination antifungal therapy with liposomal amphotericin B and voriconazole was initiated, and the PICC line was removed. Follow-up imaging revealed pulmonary consolidation and multiple hypodense lesions in the liver and spleen, consistent with disseminated fungal infection. Despite aggressive antifungal management, the patient developed persistent fungemia, multi-organ failure, and died on hospital day 81. This case underscores the diagnostic complexity, intrinsic antifungal resistance, and high mortality associated with *S. capitata*, and highlights the need for early species identification, tailored antifungal therapy, and improved fungal diagnostics in the region.

## Introduction

Invasive fungal infections are a major cause of morbidity and mortality in patients with hematological malignancies, particularly during periods of prolonged neutropenia. While *Candida* and *Aspergillus* species remain the most common culprits, emerging rare fungi have increasingly been reported in immunocompromised hosts. *Saprochaete capitata* (formerly *Geotrichum capitatum* and *Trichosporon capitatum*) is an opportunistic, ascomycetous yeast that can cause rapidly progressive and often fatal infections in patients with acute leukemia undergoing intensive chemotherapy [1-3].

Though typically non-pathogenic in immunocompetent individuals, *S.* *capitata *has been associated with bloodstream infections, pulmonary and hepatic dissemination, and deep organ involvement in neutropenic patients. It is intrinsically resistant to echinocandins and exhibits variable susceptibility to azoles, posing additional therapeutic challenges. Clinical presentation is often nonspecific, and delays in diagnosis are associated with poor outcomes, with reported case fatality rates ranging between 50% and 70% [4,5].

Although cases have been reported in Europe, Japan, and the Mediterranean region, *S.* *capitata *remains poorly characterized in the Middle East, where published data remain scarce. A few cases have been documented in the region, including the United Arab Emirates (UAE), but detailed clinical descriptions remain limited, highlighting the importance of continued reporting and awareness.

Here, we present a rare case of *S. capitata* fungemia in the UAE, occurring in a patient with acute myeloid leukemia and neutropenia, and resulting in disseminated infection and death despite timely antifungal therapy. This report highlights the organism’s emerging clinical relevance, diagnostic pitfalls, and therapeutic limitations in a region with limited fungal surveillance.

## Case presentation

A 49-year-old male with a medical history of type 2 diabetes mellitus and hypertension presented to a tertiary care hospital in the United Arab Emirates in October 2019 with a sore throat, fatigue, and unintentional weight loss. Laboratory investigations revealed marked leukocytosis (white blood cell count: 110.9 × 10⁹/L), anemia (hemoglobin: 108 g/L), and thrombocytopenia (platelet count: 135 × 10⁹/L). Contrast-enhanced thoracic CT showed bilateral axillary lymphadenopathy without pulmonary lesions. Abdominal imaging demonstrated mild hepatomegaly and extensive lymphadenopathy involving the mesenteric, peripancreatic, para-aortic, and inguinal regions.

Bone marrow aspiration and biopsy performed on hospital day one confirmed acute monoblastic leukemia, with 82% myeloid blasts. Cytogenetics were normal, and FLT3 mutation analysis revealed a low allelic ratio (0.45). A peripherally inserted central catheter (PICC) was placed, and induction chemotherapy with idarubicin and cytarabine was initiated on hospital day seven. Prophylactic antimicrobials included fluconazole, acyclovir, and piperacillin-tazobactam.

On hospital day 13 (day six of induction), the patient developed high-grade fever despite ongoing prophylaxis. Empiric coverage was escalated to meropenem and caspofungin. Serial blood cultures remained negative, and follow-up imaging did not reveal new pulmonary lesions. Due to persistent fever, vancomycin and amikacin were added on hospital day 28.

On hospital day 29, the patient acutely deteriorated, developing renal failure requiring continuous renal replacement therapy (CRRT). Blood cultures drawn from the PICC line grew yeast within 24 hours. Liposomal amphotericin B (5 mg/kg/day) was initiated, and the PICC line was removed.

The isolate was identified as* Saprochaete capitata *(formerly *Geotrichum capitatum*) using biochemical profiling and carbohydrate assimilation tests, performed via the API 20C AUX system (bioMérieux, Marcy-l'Étoile, France), which analyzes yeast metabolic patterns based on 20 carbohydrate substrates. Urease testing was negative, distinguishing it from *Trichosporon *species. Antifungal susceptibility testing (CLSI M27-A3 method) revealed sensitivity to amphotericin B (minimum inhibitory concentration (MIC) = 0.5 μg/mL) and voriconazole (MIC = 0.25 μg/mL), with high MICs to fluconazole (>64 μg/mL) and echinocandins. Voriconazole was added on hospital day 31. Additionally, no fungal biomarkers such as galactomannan or β-D-glucan were utilized during the diagnostic workup due to limited availability at our center at the time of evaluation.

**Figure 1:**
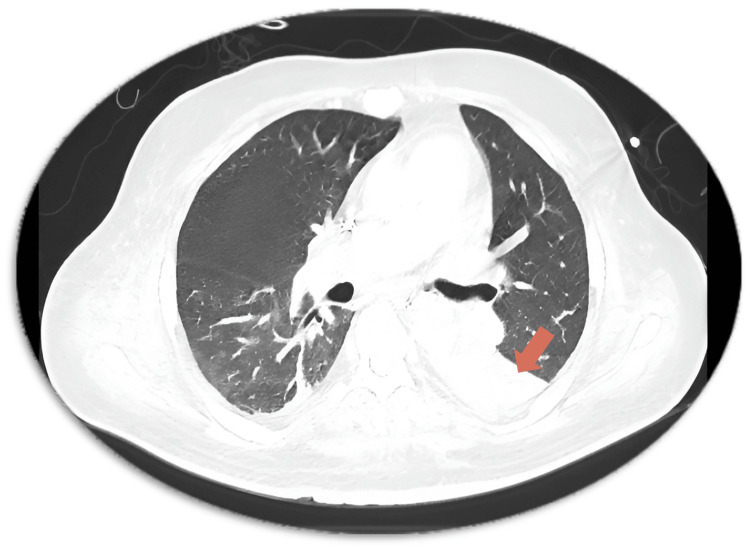
Contrast-Enhanced CT Chest on Hospital Day 32 Demonstrating Left Lower Lobe Consolidation Contrast-enhanced computed tomography (CT) of the chest performed on hospital day 32 shows dense consolidation in the left lower lobe (orange arrow), consistent with invasive fungal pneumonia in a neutropenic patient with confirmed *Saprochaete capitata *fungemia.

**Figure 2:**
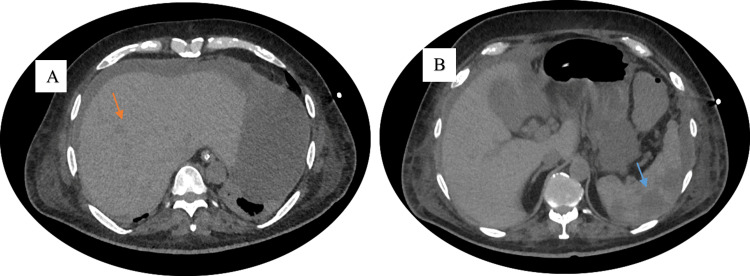
Contrast-Enhanced CT Abdomen on Hospital Day 32 Showing Hepatic and Splenic Hypodense Lesions Contrast-enhanced computed tomography (CT) of the abdomen performed on hospital day 32 reveals disseminated visceral involvement in a neutropenic patient with *Saprochaete capitata* fungemia. (A) Multiple hypodense lesions in the liver (orange arrow), suggestive of fungal microabscesses or infarcts. (B) Multiple hypodense lesions in the spleen (blue arrow), also indicative of fungal microabscesses or infarcts.

Repeat CT imaging on hospital day 32 revealed new consolidation in the left lower lobe (Figure 1) and multiple hypodense lesions in the liver and spleen (Figure 2A, 2B), consistent with disseminated fungal infection. Despite dual antifungal therapy, the patient remained febrile, and repeated blood cultures confirmed persistent fungemia.

Over the following six weeks, the patient developed progressive multi-organ dysfunction, including worsening renal, hepatic, and respiratory failure. After multidisciplinary discussions and in accordance with the family’s wishes, care was transitioned to palliation. The patient died on hospital day 81. A summary of key clinical events is presented in Table 1.

**Table 1:** Timeline of Clinical Events During Hospitalization Summary of clinical progression from admission to death in a patient with AML complicated by disseminated *Saprochaete capitata* infection. AML – Acute Myeloid Leukemia; PICC – Peripherally Inserted Central Catheter; CRRT – Continuous Renal Replacement Therapy; CT – Computed Tomography.

Hospital Day	Clinical Event
Day 1	Admission; leukocytosis, anemia, thrombocytopenia; bone marrow confirms AML
Day 2–6	Conservative management
Day 7	PICC insertion; start of idarubicin + cytarabine; prophylactic antimicrobials
Day 13	High-grade fever; escalate to meropenem + caspofungin
Day 28	Persistent fever; escalate to vancomycin + amikacin
Day 29	Acute renal failure; CRRT initiated; blood cultures grow yeast
Day 30–31	*Saprochaete capitata* ​​​​​identified; amphotericin B + voriconazole initiated; PICC removed
Day 32	CT: new left lower lobe consolidation + splenic hypodensities
Days 45–80	Persistent fungemia; progressive multi-organ failure
Day 81	Transition to palliative care; patient deceased

## Discussion

*Saprochaete capitata*, formerly classified as *Geotrichum capitatum, Trichosporon capitatum,* and *Magnusiomyces capitatus*, is a rare opportunistic fungal pathogen. It has been isolated from a variety of environmental sources, including soil and water, but can also colonize human mucosal surfaces, particularly the skin, gastrointestinal tract, and respiratory tract. These colonized sites may act as portals of entry in immunocompromised individuals, especially during episodes of mucosal barrier breakdown or broad-spectrum antibiotic use [3,4,6]. In our case, while a definitive source of infection could not be established, the presence of new pulmonary consolidation on thoracic imaging suggested the possibility of respiratory translocation as the initial site of invasion.

Microscopically, *S. capitata *is an ascomycetous yeast that produces septate, hyaline hyphae and arthroconidia. These morphological features closely resemble those of *Trichosporon *species, often leading to diagnostic confusion in routine clinical settings. However, differentiation is possible through biochemical assays, particularly carbohydrate assimilation profiles and the absence of urease activity; *S.* *capitata *is urease-negative, unlike *Trichosporon*. Such accurate identification is crucial, as therapeutic responses and antifungal susceptibility patterns differ significantly between these genera [6].

In immunocompetent individuals, *Saprochaete capitata *is generally regarded as non-pathogenic. However, it can become highly invasive in immunocompromised hosts, particularly those with hematologic malignancies. Profound and prolonged neutropenia, commonly defined as an absolute neutrophil count below 100/mm³, is the most significant predisposing factor, as it impairs innate fungal clearance. Additional risk factors include exposure to cytotoxic chemotherapy, long-term corticosteroid use, and prolonged intensive care unit (ICU) hospitalization [5,6]. Infections have also been reported in solid organ transplant recipients and patients with indwelling central venous catheters, though these cases are less frequent and often associated with coexisting immunosuppression [5,6].

The global distribution of *S.* *capitata *infections remains limited but is best documented in Europe, particularly in Mediterranean countries such as Italy, Spain, and Greece, as well as in Japan. In these regions, multiple case series have described outbreaks and sporadic infections in patients with acute leukemia. Despite the presence of similar at-risk populations and hematology practices, only a few cases have been documented in the United Arab Emirates and neighboring Gulf states [4,7], often lacking detailed clinical descriptions. This underrepresentation likely reflects underdiagnosis due to limited fungal surveillance and diagnostic infrastructure, rather than true geographic exclusion. Our report, therefore, adds to the global map of *S.* *capitata *and suggests that it may be emerging in regions previously thought to be unaffected.

A large retrospective multicenter study from Italy spanning two decades identified 52 cases of invasive *Trichosporon *infections, of which 35 were attributed to *Geotrichum capitatum *(now *Saprochaete capitata*) [4]. Among these, 65.4% of affected individuals had acute myeloid leukemia, and the estimated incidence of S. capitata infection among leukemia patients was approximately 0.5%. Positive blood cultures were reported in 76.9% of cases, pulmonary involvement in 26.9%, and the overall mortality rate was 57.1%. Another retrospective analysis similarly emphasized the predominance of *S*. capitata infections in Europe and the Mediterranean basin, where the vast majority of patients had hematological malignancies, most commonly acute leukemia [4].

Although in vitro susceptibility data consistently show that *S.* *capitata *is responsive to amphotericin B and voriconazole, the clinical efficacy of these agents remains suboptimal, with treatment failure and mortality common despite early antifungal initiation. Notably, the organism is intrinsically resistant to echinocandins and exhibits variable susceptibility to fluconazole, making empirical treatment in neutropenic patients particularly challenging. These findings highlight the urgent need for controlled therapeutic studies and standardized treatment protocols, as current clinical decisions rely largely on retrospective series and expert opinion.

Clinically, *S.* *capitata *infections are characterized by persistent high-grade fever, profound neutropenia (often agranulocytosis), and poor clinical response to empirical broad-spectrum antibiotics. Bloodstream involvement is common, and the organism is also frequently isolated from respiratory specimens such as sputum or bronchoalveolar lavage fluid. Radiological findings vary but may include halo or crescent signs, consolidations, or multiple nodular infiltrates, especially involving the lungs, liver, and spleen [8]. In our case, the constellation of persistent febrile neutropenia, new-onset pulmonary consolidation, and multiple hypodense hepatic and splenic lesions was consistent with disseminated invasive fungal disease. While no histopathological confirmation was obtained, the radiographic pattern and microbiological data provided compelling evidence for invasive *S.* *capitata *infection.

The most frequently observed clinical manifestations of *Saprochaete capitata *include mucosal lesions affecting the oral cavity and oropharynx. Diagnosis can be supported by microbiological identification of the organism through throat swab cultures or by histopathological examination of skin or mucosal biopsy specimens [9].

A MEDLINE literature review covering the period from 1965 to 2011 identified 202 published reports involving *Geotrichum, Blastoschizomyces*, and *Trichosporon capitatum*. Among these, 186 cases were classified as invasive infections, with a reported mortality rate of up to 50%. Notably, the youngest affected patient was seven years old [10]. Literature also indicates that the blood culture positivity rate for* Geotrichum capitatum* infections may reach 70%, which is significantly higher than that reported for *Candida *(<50%), *Aspergillus *(<10%), or *Fusarium *(~56%). Furthermore, deep organ involvement occurs in approximately 60-80% of patients with *G.* *capitatum *infection [10].

Early initiation of antifungal therapy is essential, as delays in treatment have been associated with mortality rates approaching 90%. Given the rarity of the infection and the limited clinical data available, no standardized therapeutic protocol has been established. Nevertheless, amphotericin B - either as monotherapy or in combination with flucytosine - is frequently used. Alternative antifungal agents such as itraconazole, voriconazole, and posaconazole have demonstrated promising results based on in vitro susceptibility testing. Importantly, *Saprochaete capitata *exhibits dose-dependent susceptibility to fluconazole [4,11].

Our case contributes to the existing literature by documenting disseminated infection confirmed through imaging, resistance to first-line azoles, and clinical failure despite dual antifungal therapy, further supporting the need for early, aggressive treatment and regional diagnostic capacity building.

## Conclusions

*Saprochaete capitata* is a rare but aggressive opportunistic fungal pathogen that presents significant diagnostic and therapeutic challenges, particularly in immunocompromised patients with hematologic malignancies. This case highlights the organism’s resistance to common antifungals, including echinocandins and fluconazole, and emphasizes the importance of rapid species identification and susceptibility testing. Despite prompt intervention, the patient developed disseminated infection and died from multi-organ failure, underscoring the pathogen’s high mortality risk. Increased clinical awareness, improved diagnostic capacity, and regional surveillance are essential to enhance early detection and outcomes for emerging fungal infections in the Middle East. The establishment of regional fungal reference laboratories, equipped with molecular and proteomic identification tools, is urgently needed to facilitate timely diagnosis and guide effective antifungal therapy.

## References

[1] Durán Graeff L, Seidel D, Vehreschild MJ (2017). Invasive infections due to Saprochaete and Geotrichum species: report of 23 cases from the FungiScope Registry. Mycoses.

[2] Martino R, Subirà M (2002). Invasive fungal infections in hematology: new trends. Ann Hematol.

[3] Vaux S, Criscuolo A, Desnos-Ollivier M (2014). Multicenter outbreak of infections by Saprochaete clavata, an unrecognized opportunistic fungal pathogen. mBio.

[4] Girmenia C, Pagano L, Martino B (2005). Invasive infections caused by Trichosporon species and Geotrichum capitatum in patients with hematological malignancies: a retrospective multicenter study from Italy and review of the literature. J Clin Microbiol.

[5] Martino R, Salavert M, Parody R (2004). Blastoschizomyces capitatus infection in patients with leukemia: report of 26 cases. Clin Infect Dis.

[6] Bouza E, Muñoz P (2004). Invasive infections caused by Blastoschizomyces capitatus and Scedosporium spp. Clin Microbiol Infect.

[7] Binder U, Lass-Flörl C (2011). Epidemiology of invasive fungal infections in the Mediterranean area. Mediterr J Hematol Infect Dis.

[8] Christakis G, Perlorentzou S, Aslanidou M, Megalakaki A, Velegraki A (2005). Fatal Blastoschizomyces capitatus sepsis in a neutropenic patient with acute myeloid leukemia: first documented case from Greece. Mycoses.

[9] Huang CL, Lu MY, Lin KH, Huang LM (2004). Geotrichum capitatum fungemia with skin lesions similar to varicella in a patient with acute lymphocytic leukemia. Acta Paediatr Taiwan.

[10] Özkaya-Parlakay A, Cengiz AB, Karadağ-Öncel E (2012). Geotrichum capitatum septicemia in a hematological malignancy patient with positive galactomannan antigen: case report and review of the literature. Turk J Pediatr.

[11] Brunetti G, Visconti V, Ghezzi MC, Mantovani S, Ferretti G, Raponi G (2016). Management and treatment of Magnusiomyces capitatus (Geotrichum capitatum) pleural infection in a non-neutropenic patient with posaconazole. A new therapeutic opportunity?. New Microbiol.

